# WHAT MAKES YOU FEEL AFRAID OF DYING: A SYSTEMATIC REVIEW OF DEATH ANXIETY AMONG KOREAN OLDER ADULTS

**DOI:** 10.1093/geroni/igae098.3768

**Published:** 2024-12-31

**Authors:** Haeun Kim, Yeonjung Lee

**Affiliations:** Chung-Ang University, Seoul, Republic of Korea; Chung-Ang University, Seoul, Republic of Korea

## Abstract

Death anxiety (DA) refers to negative emotional responses such as anxiety, fear, and denial toward death that result from the inevitability of death. Studies have suggested that older adults experience higher levels of DA than other age groups in Korea, and DA is related negatively with suicidality. Given that the suicide rate among Korean older adults is extremely high as about 39.9 suicides per 100,000 people aged 65 and older as of 2022, DA and related studies need to be appraised and synthesized. This study aims to examine recent research and provide a theoretical basis for various intervention programs by conducting a systematic literature review on factors influencing DA among Korean older people. The three key electronic databases in Korea (e-Article, KCI, and RISS) were extensively searched using the selected keywords. Among the peer-reviewed articles written in Korean since 2015, a total of 21 eligible articles were included for analysis after excluding qualitative studies, dissertations, overlapping studies, and gray literature. Findings show that the factors influencing DA can be classified as followings: sociodemographic, psychological, and medical factors, intervention programs, attitudes toward death, and preparation for death. Among the psychological factors, depression was found to relate significantly and positively with DA. Regarding the DA interventions, some programs implemented at the community such as positive psychology improvement program, dance sports, music and physical activities have a significant effect on reducing DA. Findings provide a theoretical basis for developing the intervention programs and practical strategies and thus, alleviating DA among older adults.

